# Redetermination of the borax structure from laboratory X-ray data at 145 K

**DOI:** 10.1107/S1600536808010441

**Published:** 2008-04-23

**Authors:** Graeme J. Gainsford, Tim Kemmitt, Caleb Higham

**Affiliations:** a Industrial Research Limited, PO Box 31-310, Lower Hutt, New Zealand

## Abstract

The title compound, sodium tetraborate decahydrate (mineral name: borax), Na_2_[B_4_O_5_(OH)_4_]·8H_2_O, has been studied previously using X-ray [Morimoto (1956). *Miner. J.* **2**, 1–18] and neutron [Levy & Lisensky (1978). *Acta Cryst.* B**34**, 3502–3510] diffraction data. The structure contains tetra­borate anions [B_4_O_5_(OH)_4_]^2−^ with twofold rotation symmetry, which form hydrogen-bonded chains, and [Na(H_2_O)_6_] octa­hedra that form zigzag chains [Na(H_2_O)_4/2_(H_2_O)_2/1_]. The O—H bond distances obtained from the present redetermination at 145 K are shorter than those in the neutron study by an average of 0.127 (19) Å.

## Related literature

For previous studies of the borax structure, see: Morimoto (1956 ▶); Levy & Lisenky (1978 ▶). For other structures listed in the Cambridge Structural Database (Allen, 2002 ▶) that contain the [B_4_O_5_(OH)_4_]^2−^ anion, see: Wang *et al.* (2004 ▶); Pan *et al.* (2007 ▶). For related structures, see: Yi *et al.* (2005 ▶). For comparative studies of hydrogen bonds obtained from X-ray and neutron data, see: Allen (1986 ▶); Smrčok *et al.* (2006 ▶).

## Experimental

### 

#### Crystal data

Na_2_[B_4_O_5_(OH)_4_]·8H_2_O
*M* *_r_* = 381.38
Monoclinic, 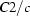

*a* = 11.8843 (5) Å
*b* = 10.6026 (4) Å
*c* = 12.2111 (5) Å
β = 106.790 (2)°
*V* = 1473.06 (10) Å^3^
*Z* = 4
Mo *K*α radiation
μ = 0.22 mm^−1^
*T* = 145 (2) K
0.65 × 0.36 × 0.26 mm

#### Data collection

Bruker–Nonius APEX2 CCD area-detector diffractometer
Absorption correction: multi-scan (*SADABS*; Bruker, 2006 ▶) *T*_min_ = 0.813, *T*_max_ = 0.94
8429 measured reflections
2275 independent reflections
2137 reflections with *I* > 2σ(*I*)
*R*_int_ = 0.018

#### Refinement

*R*[*F* ^2^ > 2σ(*F* ^2^)] = 0.025
*wR*(*F* ^2^) = 0.076
*S* = 1.08
2275 reflections
147 parameters
All H-atom parameters refined
Δρ_max_ = 0.37 e Å^−3^
Δρ_min_ = −0.22 e Å^−3^

### 

Data collection: *APEX2* (Bruker, 2006 ▶); cell refinement: *APEX2*; data reduction: *APEX2*; program(s) used to solve structure: *SHELXS97* (Sheldrick, 2008 ▶); program(s) used to refine structure: *SHELXL97* (Sheldrick, 2008 ▶); molecular graphics: *ORTEP-3* (Farrugia, 1997 ▶) and *Mercury* (Macrae *et al.*, 2006 ▶); software used to prepare material for publication: *SHELXL97* and *PLATON* (Spek, 2003 ▶).

## Supplementary Material

Crystal structure: contains datablocks global, I. DOI: 10.1107/S1600536808010441/wm2174sup1.cif

Structure factors: contains datablocks I. DOI: 10.1107/S1600536808010441/wm2174Isup2.hkl

Additional supplementary materials:  crystallographic information; 3D view; checkCIF report

## Figures and Tables

**Table 1:** Selected bond lengths (Å)

Na1—O8^i^	2.3815 (6)
Na1—O6^ii^	2.3979 (5)
Na1—O7	2.4121 (6)
Na2—O7^iii^	2.4041 (6)
Na2—O9	2.4214 (6)
Na2—O6	2.4441 (6)
B1—O4	1.4451 (8)
B1—O1	1.4657 (7)
B1—O2	1.4902 (8)
B1—O3	1.5075 (8)
B2—O2	1.3655 (8)
B2—O3^i^	1.3757 (8)
B2—O5	1.3784 (8)

Symmetry codes: (i) 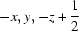; (ii) 
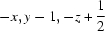
; (iii) 
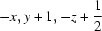
.

**Table 2:** Hydrogen-bond geometry (Å, °)

*D*—H⋯*A*	*D*—H	H⋯*A*	*D*⋯*A*	*D*—H⋯*A*
O5—H5^iv^⋯O3^iv^	0.836 (15)	1.895 (15)	2.7300 (7)	176.3 (15)
O4^v^—H4⋯O9^vi^	0.828 (14)	2.049 (14)	2.8658 (8)	168.4 (12)
O6—H6*A*^vii^⋯O5^viii^	0.868 (16)	1.978 (16)	2.8323 (8)	167.9 (14)
O6^ix^—H6*B*⋯O4^v^	0.846 (16)	2.040 (15)	2.8624 (8)	163.9 (16)
O7^vii^—H7*A*⋯O2	0.827 (16)	1.989 (16)	2.8135 (8)	174.1 (12)
O7—H7*B*⋯O4	0.816 (15)	2.135 (15)	2.9233 (8)	162.3 (14)
O8^v^—H8*A*⋯O1^x^	0.866 (13)	1.936 (13)	2.7865 (6)	167.0 (14)
O8^v^—H8*B*⋯O5^xi^	0.855 (15)	2.341 (14)	3.1320 (8)	154.2 (12)
O9—H9*A*^vii^⋯O3	0.843 (16)	2.253 (16)	3.0894 (8)	171.7 (15)
O9—H9*B*^xii^⋯O8^xiii^	0.849 (17)	2.069 (16)	2.9034 (8)	167.4 (15)

Symmetry codes: (iv) 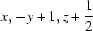; (v) 
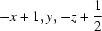
; (vi) 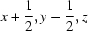; (vii) 
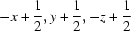
; (viii) 
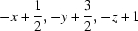
; (ix) 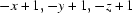; (x) 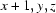; (xi) 
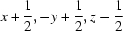
; (xii) 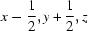; (xiii) 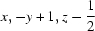.

**Fig. 1.**
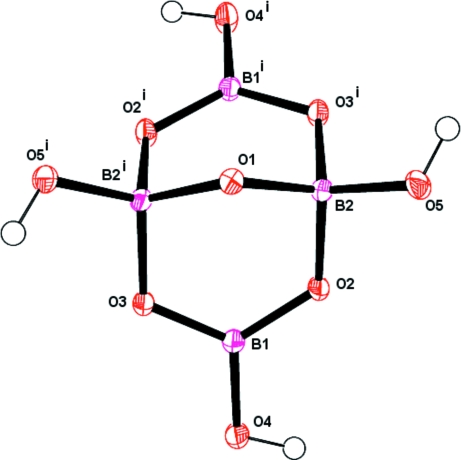
Molecular structure of the tetraborate anion shown with displacement ellipsoids at the 50% probability level. [Symmetry code: i) -x, y, 1/2 - z.]

**Fig. 2.**
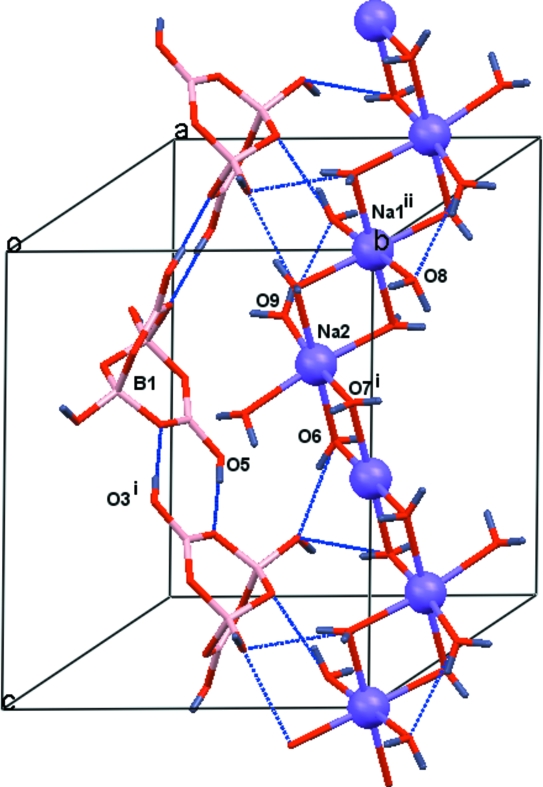
Part of the crystal structure showing the zigzag [Na(H2O)4/2(H2O)2/1] chain, the hydrogen bonded tetraborate chain and some interlinking hydrogen bonds, shown as dashed lines. For clarity, only selected atoms and one of each chain is shown. [Symmetry codes: i) -x, y 1/2 - z; ii) x, 1 + y, z.]

## supplementary crystallographic information

### Comment

The crystal structure of the title compound was previously studied by Morimoto (1956) using X-ray and later by Levy & Lisensky (1978, hereafter LL) using neutron diffraction data.

There are 8 other compounds with free tetraborate anions [B_4_O_5_(OH)_4_]^2-^ reported in the Cambridge Structural Database [C.S.D., version 5.29 with November 2007 updates (Allen, 2002)] with most containing protonated amine-based cations, *e.g.* DALQEN (Wang *et al.*, 2004) and SIBDIR (Pan *et al.*, 2007). The tetraborate anion in borax has 2-fold symmetry with the axis passing through O1 (Fig. 1) as is observed in five of the related structures. Both Na1 and Na2 cations are on special positions (centre of symmetry and 2-fold axis, respectively) so that they elegantly bind *via* shared water molecules in a typical zigzag cationic chain [Na(H_2_O)_4/2_(H_2_O)_2/1_] parallel to the *c* axis (*e.g.* DARNOA, Yi *et al.*, 2005), as shown in Figure 2. As is found through a C.S.D. search of similar Na^+^/H_2_O cation chains, the Na–O distances to the bridging water molecules are longer than those to non-bridging water molecules, where the *trans* related Na–O distances belong to non-bridging water molecules.

The results of the present study and the LL model are essentially superimposable, but do reflect expected differences associated with the H atom positions: The systematic pairwise study (Allen, 1986) gave a difference for O–H (X-ray versus neutron) of -0.155 (10) Å, while a more recent study of levoglucosan (Smrčok *et al.*, 2006) averaged at -0.016 (6) Å. The mean O—H distance here (0.843 (17) Å) is significantly shorter than for the neutron set (0.97 (1) Å). As the O···O distances involved in the hydrogen bonding are very similar for both studies (Table 1), the observed H···O distances are correspondingly longer here than in the LL model. We also note that average Na–O distances are marginally longer (0.006 (6) Å) and the B–O distances marginally shorter (-0.005 (2) Å) in the LL model, *e.g.* Na–O6, B1–O2 are 2.458 (3), 1.500 (2) Å compared with 2.4441 (6), 1.5075 (8) Å, respectively, in the present study. These latter differences are barely significant given that the neutron data set was collected at 296.5 K.

Cell cohesion is provided by strong O—H···O hydrogen bonds of two types: (1) tetraborate anions "head to tail" link *via* the O5–H and O2 atoms (entry 1, Table 1) to form anionic chains as also seen in DALQEN (Wang *et al.*, 2004); (2) the anionic and cationic chains crosslink through the water & tetraborate strong O–H···O hydrogen bond interactions (entries 2–10; see also Fig. 2 and diagrams in the LL study).

### Experimental

To a tetrahydrofuran (thf) solution (90 ml) of sodium tetrahydridoborate (0.31 g, 8.4 mmol) was added 0.5 g (4.2 mmol) of diaminomethane dihydrochloride. After 24 h, the solvent was removed and the remaining product dissolved in water. Methanol was added and the solution was left in a refrigerator. A small clump of colourless crystals of the title compound appeared after several days in the bottom of the flask.

### Refinement

A total of 13 reflections (below 50°/2θ) were not collected. In the present re-determination the same atomic labels and atomic coordinates have been used as in the previous studies (Morimoto, 1956; Levy & Lisensky, 1978). The positions of the H atoms were fully refined with isotropic thermal parameters for each H atom.

### Crystal data

**Table:**

Na_2_[B_4_O_5_(OH)_4_]·8H_2_O	*F*_000_ = 792
*M**_r_* = 381.38	*D*_x_ = 1.720 Mg m^−^^3^
Monoclinic, *C*2/*c*	Mo *K*α radiation λ = 0.71073 Å
Hall symbol: -C 2yc	Cell parameters from 6688 reflections
*a* = 11.8843 (5) Å	θ = 2.6–31.9º
*b* = 10.6026 (4) Å	µ = 0.22 mm^−^^1^
*c* = 12.2111 (5) Å	*T* = 145 (2) K
β = 106.790 (2)º	Prism, colourless
*V* = 1473.06 (10) Å^3^	0.65 × 0.36 × 0.26 mm
*Z* = 4	

### Data collection

**Table:**

Bruker–Nonius APEX2 CCD area-detector diffractometer	2275 independent reflections
Radiation source: fine-focus sealed tube	2137 reflections with *I* > 2σ(*I*)
Monochromator: graphite	*R*_int_ = 0.018
Detector resolution: 8.192 pixels mm^-1^	θ_max_ = 33.2º
*T* = 145(2) K	θ_min_ = 2.6º
φ and ω scans	*h* = −17→16
Absorption correction: multi-scan(SADABS; Bruker, 2006)	*k* = −15→15
*T*_min_ = 0.813, *T*_max_ = 0.94	*l* = −16→17
8429 measured reflections	

### Refinement

**Table:**

Refinement on *F*^2^	Secondary atom site location: difference Fourier map
Least-squares matrix: full	Hydrogen site location: inferred from neighbouring sites
*R*[*F*^2^ > 2σ(*F*^2^)] = 0.025	All H-atom parameters refined
*wR*(*F*^2^) = 0.076	*w* = 1/[σ^2^(*F*_o_^2^) + (0.0482*P*)^2^ + 0.3901*P*] where *P* = (*F*_o_^2^ + 2*F*_c_^2^)/3
*S* = 1.08	(Δ/σ)_max_ = 0.001
2275 reflections	Δρ_max_ = 0.37 e Å^−^^3^
147 parameters	Δρ_min_ = −0.22 e Å^−^^3^
Primary atom site location: structure-invariant direct methods	Extinction correction: none

### Special details

**Table:**

Geometry. All esds (except the esd in the dihedral angle between two l.s. planes) are estimated using the full covariance matrix. The cell esds are taken into account individually in the estimation of esds in distances, angles and torsion angles; correlations between esds in cell parameters are only used when they are defined by crystal symmetry. An approximate (isotropic) treatment of cell esds is used for estimating esds involving l.s. planes.
Refinement. Refinement of *F*^2^ against ALL reflections. The weighted *R*-factor *wR* and goodness of fit *S* are based on *F*^2^, conventional *R*-factors *R* are based on *F*, with *F* set to zero for negative *F*^2^. The threshold expression of *F*^2^ > σ(*F*^2^) is used only for calculating *R*-factors(gt) *etc*. and is not relevant to the choice of reflections for refinement. *R*-factors based on *F*^2^ are statistically about twice as large as those based on *F*, and *R*- factors based on ALL data will be even larger.

### Fractional atomic coordinates and isotropic or equivalent isotropic displacement parameters (Å^2^)

**Table:**

	*x*	*y*	*z*	*U*_iso_*/*U*_eq_	
Na1	0.0000	0.0000	0.0000	0.01676 (10)	
Na2	0.0000	0.84795 (4)	0.2500	0.01796 (11)	
B1	0.08552 (6)	0.34499 (6)	0.21553 (5)	0.01044 (13)	
B2	0.09847 (6)	0.45643 (6)	0.39269 (6)	0.01154 (13)	
O1	0.0000	0.26659 (6)	0.2500	0.01094 (13)	
O2	0.15546 (4)	0.41927 (4)	0.31574 (4)	0.01276 (11)	
O3	0.01964 (4)	0.43573 (4)	0.12445 (4)	0.01339 (11)	
O4	0.16140 (4)	0.27014 (5)	0.16772 (4)	0.01570 (11)	
O5	0.16369 (4)	0.51522 (5)	0.49130 (4)	0.01815 (12)	
O6	0.12357 (5)	0.84607 (5)	0.44846 (5)	0.01851 (12)	
O7	0.12296 (5)	0.00117 (5)	0.19548 (5)	0.01799 (12)	
O8	0.11919 (5)	0.16556 (5)	0.46252 (5)	0.02067 (12)	
O9	0.11746 (5)	0.70654 (6)	0.17227 (5)	0.02187 (12)	
H4	0.7717 (12)	0.2622 (11)	0.2876 (12)	0.032 (3)*	
H5	0.1187 (13)	0.4667 (13)	0.0305 (12)	0.040 (3)*	
H6A	0.3089 (13)	0.3828 (14)	0.0413 (12)	0.042 (4)*	
H6B	0.8662 (14)	0.2018 (16)	0.4941 (13)	0.051 (4)*	
H7A	0.3098 (13)	0.4817 (11)	0.3051 (12)	0.030 (3)*	
H7B	0.1304 (12)	0.0776 (14)	0.2014 (12)	0.039 (3)*	
H8A	0.9099 (12)	0.1906 (13)	0.1075 (11)	0.036 (3)*	
H8B	0.8131 (12)	0.1365 (12)	0.0352 (11)	0.034 (3)*	
H9A	0.4018 (13)	0.1300 (15)	0.3385 (12)	0.046 (4)*	
H9B	0.6140 (15)	0.2331 (15)	0.1058 (14)	0.053 (4)*	

### Atomic displacement parameters (Å^2^)

**Table:**

	*U*^11^	*U*^22^	*U*^33^	*U*^12^	*U*^13^	*U*^23^
Na1	0.0165 (2)	0.01812 (19)	0.0158 (2)	0.00057 (13)	0.00485 (15)	0.00047 (13)
Na2	0.0179 (2)	0.0201 (2)	0.0176 (2)	0.000	0.00773 (16)	0.000
B1	0.0100 (3)	0.0121 (3)	0.0096 (3)	0.0012 (2)	0.0035 (2)	−0.00028 (19)
B2	0.0108 (3)	0.0133 (3)	0.0111 (3)	−0.0014 (2)	0.0040 (2)	−0.0014 (2)
O1	0.0117 (3)	0.0104 (3)	0.0111 (3)	0.000	0.0037 (2)	0.000
O2	0.0103 (2)	0.0168 (2)	0.0122 (2)	−0.00223 (15)	0.00493 (16)	−0.00376 (15)
O3	0.0103 (2)	0.0180 (2)	0.0130 (2)	0.00309 (15)	0.00519 (16)	0.00559 (15)
O4	0.0119 (2)	0.0209 (2)	0.0142 (2)	0.00489 (17)	0.00365 (17)	−0.00434 (16)
O5	0.0132 (2)	0.0275 (3)	0.0150 (2)	−0.00595 (18)	0.00593 (19)	−0.00949 (18)
O6	0.0146 (2)	0.0219 (2)	0.0186 (2)	−0.00109 (18)	0.00414 (19)	0.00229 (18)
O7	0.0151 (3)	0.0160 (2)	0.0232 (3)	0.00012 (17)	0.0061 (2)	0.00003 (17)
O8	0.0185 (3)	0.0242 (3)	0.0184 (3)	0.00116 (19)	0.0039 (2)	0.00608 (19)
O9	0.0217 (3)	0.0196 (2)	0.0229 (3)	−0.00047 (19)	0.0041 (2)	−0.00352 (19)

### Geometric parameters (Å, °)

**Table:**

Na1—O8^i^	2.3815 (6)	Na2—O6^ii^	2.4441 (6)
Na1—O8^ii^	2.3815 (6)	Na2—O6	2.4441 (6)
Na1—O6^iii^	2.3979 (5)	B1—O4	1.4451 (8)
Na1—O6^iv^	2.3979 (5)	B1—O1	1.4657 (7)
Na1—O7^v^	2.4121 (6)	B1—O2	1.4902 (8)
Na1—O7	2.4121 (6)	B1—O3	1.5075 (8)
Na2—O7^vi^	2.4041 (6)	B2—O2	1.3655 (8)
Na2—O7^vii^	2.4041 (6)	B2—O3^ii^	1.3757 (8)
Na2—O9	2.4214 (6)	B2—O5	1.3784 (8)
Na2—O9^ii^	2.4214 (6)		
			
O8^i^—Na1—O8^ii^	180.00 (2)	O9—Na2—O6^ii^	81.696 (19)
O8^i^—Na1—O6^iii^	90.45 (2)	O9^ii^—Na2—O6^ii^	97.72 (2)
O8^ii^—Na1—O6^iii^	89.55 (2)	O7^vi^—Na2—O6	88.29 (2)
O8^i^—Na1—O6^iv^	89.55 (2)	O7^vii^—Na2—O6	92.34 (2)
O8^ii^—Na1—O6^iv^	90.45 (2)	O9—Na2—O6	97.72 (2)
O6^iii^—Na1—O6^iv^	180.00 (3)	O9^ii^—Na2—O6	81.70 (2)
O8^i^—Na1—O7^v^	91.717 (19)	O6^ii^—Na2—O6	179.07 (3)
O8^ii^—Na1—O7^v^	88.283 (19)	O4—B1—O1	111.72 (5)
O6^iii^—Na1—O7^v^	89.177 (19)	O4—B1—O2	110.91 (5)
O6^iv^—Na1—O7^v^	90.823 (19)	O1—B1—O2	109.42 (4)
O8^i^—Na1—O7	88.283 (19)	O4—B1—O3	107.71 (5)
O8^ii^—Na1—O7	91.717 (19)	O1—B1—O3	108.56 (5)
O6^iii^—Na1—O7	90.823 (19)	O2—B1—O3	108.43 (5)
O6^iv^—Na1—O7	89.177 (19)	O2—B2—O3^ii^	122.44 (6)
O7^v^—Na1—O7	180.000 (16)	O2—B2—O5	117.78 (6)
O7^vi^—Na2—O7^vii^	94.98 (3)	O3^ii^—B2—O5	119.78 (6)
O7^vi^—Na2—O9	172.90 (2)	B1^ii^—O1—B1	110.90 (6)
O7^vii^—Na2—O9	81.05 (2)	B2—O2—B1	116.59 (5)
O7^vi^—Na2—O9^ii^	81.05 (2)	B2^ii^—O3—B1	120.25 (5)
O7^vii^—Na2—O9^ii^	172.90 (2)	Na1^vi^—O6—Na2	90.952 (19)
O9—Na2—O9^ii^	103.49 (3)	Na2^viii^—O7—Na1	91.581 (19)
O7^vi^—Na2—O6^ii^	92.34 (2)	Na2^viii^—O7—H7B	134.8 (10)
O7^vii^—Na2—O6^ii^	88.29 (2)	Na1—O7—H7B	96.4 (10)
			
O4—B1—O1—B1^ii^	−172.74 (6)	O7^vi^—Na2—O6—Na1^vi^	−0.355 (19)
O2—B1—O1—B1^ii^	64.05 (4)	O7^vii^—Na2—O6—Na1^vi^	94.56 (2)
O3—B1—O1—B1^ii^	−54.12 (3)	O9—Na2—O6—Na1^vi^	175.84 (2)
O3^ii^—B2—O2—B1	−4.91 (9)	O9^ii^—Na2—O6—Na1^vi^	−81.56 (2)
O5—B2—O2—B1	174.23 (5)	O6^ii^—Na2—O6—Na1^vi^	−132.911 (15)
O4—B1—O2—B2	−156.88 (5)	O8^i^—Na1—O7—Na2^viii^	−89.93 (2)
O1—B1—O2—B2	−33.19 (7)	O8^ii^—Na1—O7—Na2^viii^	90.07 (2)
O3—B1—O2—B2	85.06 (6)	O6^iii^—Na1—O7—Na2^viii^	179.640 (19)
O4—B1—O3—B2^ii^	136.95 (6)	O6^iv^—Na1—O7—Na2^viii^	−0.360 (19)
O1—B1—O3—B2^ii^	15.82 (7)	O7^v^—Na1—O7—Na2^viii^	−122 (44)
O2—B1—O3—B2^ii^	−102.97 (6)		

Symmetry codes: (i) *x*, −*y*, *z*−1/2; (ii) −*x*, *y*, −*z*+1/2; (iii) *x*, −*y*+1, *z*−1/2; (iv) −*x*, *y*−1, −*z*+1/2; (v) −*x*, −*y*, −*z*; (vi) −*x*, *y*+1, −*z*+1/2; (vii) *x*, *y*+1, *z*; (viii) *x*, *y*−1, *z*.

### Hydrogen-bond geometry (Å, °)

**Table:**

*D*—H···*A*	*D*—H	H···*A*	*D*···*A*	*D*—H···*A*
O5—H5^ix^···O3^ix^	0.836 (15)	1.895 (15)	2.7300 (7)	176.3 (15)
O4^x^—H4···O9^xi^	0.828 (14)	2.049 (14)	2.8658 (8)	168.4 (12)
O6—H6A^xii^···O5^xiii^	0.868 (16)	1.978 (16)	2.8323 (8)	167.9 (14)
O6^xiv^—H6B···O4^x^	0.846 (16)	2.040 (15)	2.8624 (8)	163.9 (16)
O7^xii^—H7A···O2	0.827 (16)	1.989 (16)	2.8135 (8)	174.1 (12)
O7—H7B···O4	0.816 (15)	2.135 (15)	2.9233 (8)	162.3 (14)
O8^x^—H8A···O1^xv^	0.866 (13)	1.936 (13)	2.7865 (6)	167.0 (14)
O8^x^—H8B···O5^xvi^	0.855 (15)	2.341 (14)	3.1320 (8)	154.2 (12)
O9—H9A^xii^···O3	0.843 (16)	2.253 (16)	3.0894 (8)	171.7 (15)
O9—H9B^xvii^···O8^iii^	0.849 (17)	2.069 (16)	2.9034 (8)	167.4 (15)

Symmetry codes: (ix) *x*, −*y*+1, *z*+1/2; (x) −*x*+1, *y*, −*z*+1/2; (xi) *x*+1/2, *y*−1/2, *z*; (xii) −*x*+1/2, *y*+1/2, −*z*+1/2; (xiii) −*x*+1/2, −*y*+3/2, −*z*+1; (xiv) −*x*+1, −*y*+1, −*z*+1; (xv) *x*+1, *y*, *z*; (xvi) *x*+1/2, −*y*+1/2, *z*−1/2; (xvii) *x*−1/2, *y*+1/2, *z*; (iii) *x*, −*y*+1, *z*−1/2.
